# The Use of Spatial Analysis in Syphilis-Related Research: Protocol for a Scoping Review

**DOI:** 10.2196/43243

**Published:** 2023-04-25

**Authors:** Janmilli da Costa Dantas, Rayssa Horacio Lopes, Cristiane da Silva Ramos Marinho, Yago Tavares Pinheiro, Richardson Augusto Rosendo da Silva

**Affiliations:** 1 Department of Nursing Faculty of Health Sciences of Trairi Federal University of Rio Grande do Norte Santa Cruz Brazil; 2 School Department of Health Federal University of Rio Grande do Norte Natal Brazil; 3 Physiotherapy Department Santa Maria University Center Cajazeiras Brazil; 4 Department of Nursing Federal University of Rio Grande do Norte Natal Brazil

**Keywords:** treponema pallidum, syphilis, infectious diseases, spatial distribution, systems of geographic information, spatiotemporal analysis, health care, surveillance, spatial analysis, geographic information, policy maker

## Abstract

**Background:**

Latin America, Africa, and Asia have high incidences of syphilis. New approaches are needed to understand and reduce disease transmissibility. In health care, spatial analysis is important to map diseases and understand their epidemiologic aspects.

**Objective:**

The proposed scoping review will identify and map the use of spatial analysis as a tool for syphilis-related research in health care.

**Methods:**

This protocol was based on the Joanna Briggs Institute manual, guided by the Preferred Reporting Items for Systematic Reviews and Meta-Analyses Extension for Scoping Reviews (PRISMA-ScR). We will conduct searches in Embase; Lilacs, via the Virtual Health Library (Biblioteca Virtual en Salud; BVS), in Portuguese and English; Medline/PubMed; Web of Science; Cumulative Index to Nursing and Allied Health Literature (CINAHL); and Scopus. Gray literature will be searched for in Google Scholar, the Digital Library of Theses and Dissertations, the Catalog of Theses and Dissertations of the Coordination of Improvement of Higher Education Personnel (Coordenação de Aperfeiçoamento de Pessoal de Nível Superior; CAPES), Open Access Theses and Dissertations, ProQuest Dissertations and Theses Global, and the Networked Digital Library of Theses and Dissertations. The main research question is “How has spatial analysis been used in syphilis-related research in health care?” Studies are included if they have the full text available, address syphilis, and use geographic information systems software and spatial analysis techniques, regardless of sample characteristics or size. Studies published as research articles, theses, dissertations, and government documents will also be considered, with no location, time, or language restrictions. Data will be extracted using a spreadsheet adapted from the Joanna Briggs Institute. Quantitative and qualitative data will be analyzed using descriptive statistics and a thematic analysis, respectively.

**Results:**

The results will be presented according to the PRISMA-ScR guidelines and will summarize the use of spatial analysis in syphilis-related research in health care in countries with different contexts, factors associated with spatial cluster formation, population health impacts, contributions to health systems, challenges, limitations, and possible research gaps. The results will guide future research and may be useful for health and safety professionals, managers, public policy makers, the general population, the academic community, and health professionals who work directly with people with syphilis. Data collection is projected to start in June 2023 and end in July 2023. Data analysis is scheduled to take place in August and September 2023. We expect to publish results in the final months of 2023.

**Conclusions:**

The review may reveal where syphilis incidence has the highest incidence, which countries most use spatial analysis to study syphilis, and whether spatial analysis is applicable to syphilis in each continent, thereby contributing to discussion and knowledge dissemination on the use of spatial analysis as a tool for syphilis-related research in health care.

**Trial Registration:**

Open Science Framework CNVXE; https://osf.io/cnvxe

**International Registered Report Identifier (IRRID):**

PRR1-10.2196/43243

## Introduction

Spatial analyses are techniques performed on geographic data that describe an entire geographic region or a single point in space [1] to measure, interpret, and explore characteristics and associations [2]. These analyses are an important tool in health care for mapping diseases and understanding their epidemiology [3,4].

The evolution of computer science has improved and expanded software development, increasing the applicability and accessibility of spatial analysis [2]. Visualizing geographic areas has enabled the mapping of disease distribution, risks and correlated factors, and the physical and human structures of health services. Spatial analysis is a useful tool to visualize areas with the greatest epidemiological pressure and infer associations between the studied phenomenon and sociopolitical and economic factors that contribute to it [5,6]. Therefore, spatial analysis contributes to planning, implementing, and assessing global health policies [3,4].

In the context of health care, it is equally important to consider the location and characteristics of where people seek care. Thus, spatial epidemiology has evolved rapidly in recent years. Research has developed to include geocoding, distance estimation, residential mobility, record linking, data integration, spatial and spatiotemporal clustering, small area estimation, and Bayesian applications for disease mapping. Computer-based geographic information systems for integrating and analyzing geographic data are applicable in epidemiology, contributing, for example, to disease mapping, rate smoothing, cluster or hot spot analysis, and spatial modeling [2].

Documenting the role of the geographic environment where individuals live and interact (often called “activity spaces”) will improve our understanding of health outcomes. This is because the place where an individual lives or works can be considered as a potential determinant of disease; this has profound political implications for local health interventions and resource-allocation decisions and will ultimately lead to a reduction in health disparities [2].

Previous research has used spatial analysis to map disease distribution (eg, for multiple sclerosis [7], HIV [8], and COVID-19 [9]), and spatial analysis could also guide health strategies against syphilis [10], which has a high incidence and represents a problem for public health worldwide, especially in Latin America, Africa, and Asia [11,12].

Syphilis spreads via sexual or vertical transmission. It can be asymptomatic, can manifest as spots on hands and feet, and can affect the functioning of several organs (in the systemic phase). Gestational syphilis and congenital syphilis directly impact reproductive and child health. During pregnancy, syphilis may lead to abortion, stillbirth, premature birth, neonatal death, and early or late congenital manifestations [13].

A previous study used spatial analysis to assess the epidemiological status of congenital syphilis in Brazil and found that the most affected municipalities were those with a large migratory flow, those bordering other countries, and those with many tourists [10]. Clusters of congenital syphilis were even identified in municipalities with prenatal care [14].

In China, researchers reported spatiotemporal changes in the incidence of syphilis; eastern coastal provinces showed a declining trend, whereas inland provinces showed an increasing trend, suggesting an association between epidemiological and economic issues [15]. In Italy, a study pointed out that women diagnosed with syphilis were more likely to be non-Italian, while men were more likely to be Italian [16]. Strategies to provide support services for accessing treatment have been implemented in Colombia for HIV and syphilis testing among Venezuelan migrants [17]. In Africa, the implementation of self-tests for HIV and syphilis in men’s workplaces can be thought of as an approach to increase the availability of and access to tests among men and provide linkage to treatment and prevention services [18].

New strategies are needed to reduce syphilis transmission, such as spatial analysis, which has previously been applied in syphilis-related research [10,14,15,19]. However, due to a lack of studies, how and to what extent this tool is being used is unclear. A search was conducted in July 2022 of the Joanna Briggs Institute, Cochrane Library, Web of Science, PubMed, Prospero, and Open Science Framework databases. The search found no review studies or protocols that used spatial analysis in syphilis-related research, highlighting this knowledge gap.

A scoping review identifying and globally mapping the application of spatial analysis in syphilis-related research would help researchers, managers, and health policy makers manage methodologies for disease control. Thus, this study describes a protocol for a scoping review to identify and map the use of spatial analysis in syphilis-related research in health care.

## Methods

### Overview

This is a protocol for a scoping review to answer broad research questions based on defined selection criteria [20]. We used the guidelines of the *Joanna Briggs Institute Reviewer’s Manual* [20], based on the theoretical framework proposed by Arkey and O’Malley [21], updated by Levac et al [22], and guided by the Preferred Reporting Items for Systematic Reviews and Meta-Analyses Extension for Scoping Reviews (PRISMA-ScR) [23].

The stages of the scoping review include (1) definition and alignment of research objectives and questions; (2) development and alignment of inclusion criteria; (3) description of the search for evidence, selection and extraction of data, and presentation of evidence; (4) the search for evidence; (5) selection of evidence; (6) extraction of evidence; (7) analysis of evidence; (8) presentation of the results; and (9) a summary of the evidence, conclusions, and the implications of the findings [20].

### Stage 1: Definition and Alignment of Research Objectives and Questions

The mnemonic PCC (population, concept, context) underlay formulation of the research question. This model allows broadly mapping information to identify knowledge gaps, present key concepts, quantify aspects of interest, and expose practices and evidence of a particular theme [20]. We defined the population as people diagnosed with syphilis, the concept as spatial analysis, and the context as health care. Thus, the research question is “How has spatial analysis been used in syphilis-related research in health care?”

### Stage 2: Development and Alignment of Inclusion Criteria According to Research Objectives and Questions

Studies will be included if the full text is available, they address syphilis, and they use any geographic information systems software and spatial analysis technique. Studies will be included independently of sample characteristics and size. Studies published as research articles, theses, dissertations, and government documents will be considered with no location, time, or language restrictions. An external translator will be used when needed.

Studies will be excluded if they are literature reviews, debates, documents, editorials, expert opinions, comments, opinion articles, or conference or poster abstracts. Studies that do not have syphilis as the primary outcome, have an insufficient methodological description, or do not answer the research question will also be excluded.

### Stage 3: Description of the Search for Evidence, Selection, Extraction of Data, and Presentation of Evidence

The search strategy will be conducted in 3 steps to reach the largest number of publications possible.

#### First Step: Identification of Descriptors and Keywords

An exploratory search on PubMed and the Virtual Health Library (Biblioteca Virtual en Salud; BVS) was conducted to identify the main medical subject heading (MeSH) terms and health sciences descriptors (Descritores em Ciências da Saúde; DeCS) related to the topic. The search strategy was built using 4 controlled health vocabularies, including DeCS, MeSH, Emtree, and the Tesauro Cumulative Index to Nursing and Allied Health Literature (CINAHL), to identify relevant studies and expand the results in different databases.

A search was conducted to identify synonyms and keywords. The search strategy was then expanded, checked, and improved by a librarian. Descriptors were combined with natural language [24,25] to increase sensitivity and expand the search results. The construction of the search strategy used the extraction, conversion, combination, construction, and use model [24]. This model increases the sensitivity of the search strategy by following complementary steps. Table 1 shows the conversion of mnemonic elements into the main keywords.

Multimedia Appendix 1 shows the complete search strategy built for Medline/PubMed.

**Table 1:** Definition of the PCC (population, concept, context) mnemonic elements.

Mnemonic elements	Definition	Keywords
Population	People diagnosed with syphilis	Syphilis; treponema pallidum; treponema infections
Concept	Spatial analysis	Spatial analysis; geographic information system; geographic information systems; disease hotspot; spatiotemporal analysis; spatiotemporal analysis; geographic mapping
Context	Health care	Identification by document reading

#### Second Step: Database Definition for Data Collection

After defining a search strategy with high sensitivity, data collection will be performed in the following databases: Embase, Lilacs (in Portuguese and English) via the BVS, Medline/PubMed, Web of Science, CINAHL, and Scopus.

For a gray literature search, we will consult Google Scholar, the Digital Library of Theses and Dissertations, the Catalog of Theses and Dissertations from the Coordination of Improvement of Higher Education Personnel (Coordenação de Aperfeiçoamento de Pessoal de Nível Superior; CAPES), Open Access Theses and Dissertations, ProQuest Dissertations and Theses Global, and the Networked Digital Library of Theses and Dissertations.

#### Third Step: Browse Additional Sources in Selected Publication References

The reference lists of selected articles will be searched to track down eligible sources not retrieved with the search strategy. Authors of the included studies will be consulted by email for any additional information if needed.

### Stage 4: Search for Evidence

The search strategy will be adapted to each database. The free version of Rayyan (Qatar Foundation) [26] will be used for duplicate removal and study selection. Two reviewers (JdCD and YTP) will conduct a pilot test to reduce biases, standardize the selection process, and verify agreement with the study protocol. Each author will select 25 studies and read the titles and abstracts; the screening will follow the eligibility criteria. The team will then discuss any discrepancies and make necessary changes to the criteria and definitions. The screening will start only after 75% agreement is reached [20].

After the pilot test, 2 blind reviewers (JdCD and YTP) will use Rayyan [26] to read the titles and abstracts of all identified studies, evaluating them according to the inclusion criteria. In cases of disagreement, a third reviewer (RARdS) will be consulted.

### Stage 5: Selection of Evidence

After reading the full text, potentially eligible publications will be retrieved in full via their titles and abstracts and exported to a Microsoft Excel database (2020 version; Microsoft Corp). Full texts will be analyzed and reasons for exclusion will be recorded. Information regarding the selection of publications, eligibility criteria, and reasons for inclusion and exclusion will be reported in the PRISMA-ScR flowchart [27].

### Stage 6: Extraction of Evidence

Data will be extracted using a spreadsheet built in Microsoft Excel (Table 2). Two trained reviewers (JdCD and YTP) will extract the data.

Two reviewers will map the application of spatial analysis in syphilis-related research using geographic data and by identifying where the study was performed. The map will be developed using a Google spreadsheet.

**Table 2:** Data extraction variables.

Variable	Standardized method
First author and year of publication	Identify the first author and year of publication of the study
Objective	Detail the study objective
Study design	Detail the study design described by the author or authors
Data collection procedures	List the data collection technique or techniques used
Geographic extent	Identify the city, state, region, or country where the study was conducted
Syphilis, gestational syphilis, congenital syphilis	Identify the classification of syphilis
Period (in years) of the collected data	Identify the period of data collection
Visualization software	Identify the geographic visualization software
Spatial analysis method	Describe the methodology developed in the spatial analysis
Results	Describe the main study results
Challenges and limitations	Describe the main study challenges and limitations mentioned by the authors related to using spatial analysis

### Stage 7: Analysis of Evidence

Results will be interpreted qualitatively and quantitatively and presented using a PRISMA-ScR flowchart [27]. SPSS (version 24.0; IBM Corp) will be used for statistical analysis. The quantitative analysis will use descriptive statistics with the absolute frequency and percentage. Thematic analysis will be used in the qualitative analysis due to its flexibility in identifying patterns in research questions [28,29]. A thematic analysis will categorize the application of spatial analysis in syphilis-related research in health care. The results will be discussed using the literature and research objectives and questions.

### Stage 8: Presentation of Results

The final report guided by PRISMA-ScR will include the results in flowcharts, graphs, or figures [23]. We will use images to help readers understand the results of the studies. As the theme of the presented protocol involves spatial analysis, it is very likely that some of the results will be shown in map images.

### Stage 9: Summary of Evidence, Conclusions, and Implications of Findings

After carrying out the previous steps, we will prepare a summary of the results of the scoping review linked to the objective of the study. This way, the conclusion of the study will be grounded and presented effectively. We will highlight knowledge gaps that may arise in the development of the scoping review to provide direction for future studies.

## Results

This protocol will guide a scoping review to identify and map the use of spatial analysis as a tool in syphilis-related research in health care. The results will be presented according to the PRISMA-ScR guidelines [23] and summarized regarding (1) the use of spatial analysis in syphilis-related research in health care in countries with different contexts, (2) factors associated with the formation of spatial clusters, and (3) impacts on population health; contributions to health systems, challenges, limitations, and possible research gaps will also be summarized to guide future research. In addition, the results of this scoping review may be useful for public health and safety professionals, managers, public policy makers, the general population, the academic community, and health professionals who work directly with people with syphilis.

This protocol will enable method replication following the principles of open science [30], minimizing the risk of bias.

Data collection is projected to start in June 2023 and end in July 2023. Data analysis is scheduled to take place in August and September 2023. We expect to publish the results of the scoping review in the final months of 2023.

## Discussion

### Principal Findings

This protocol will guide a scoping review to identify and map the use of spatial analysis as a tool in syphilis-related research in health care. Spatial analysis in health care research can identify the spatial distribution of high-incidence diseases, identify associations in their distribution with determining factors, and support direct health planning and actions [31-33].

The United Nations proposed the elimination of congenital syphilis by 2030, reducing its incidence to 0.5 cases per 1000 live births [34]. Strategies to prevent syphilis are a concern of authorities worldwide and are included in the sustainable development goals. Therefore, this review will help develop specific health strategies to control syphilis.

The research team involved in this protocol has experience studying syphilis and spatial analysis and has knowledge and experience in scoping reviews. A librarian helped develop the high-sensitivity search strategy, which is based on a combination of 4 vocabularies and will expand the results and allow access to the literature; an especially important point is the lack of time and language restrictions.

A test search with terms for epidemiology, treatment, prevalence, and incidence was performed in the thematic context group (on the use of spatial analysis in health care). However, the search had restricted results and directed the research question to a certain type of technique, creating bias. Thus, we left the “context” field empty and searched for all possibilities for using spatial analysis in health care.

### Limitations

The study limitations will include the lack of a search for institutional sites in all countries (ie, for gray literature at these sites), but this should not affect study development. We will select essential databases to minimize this limitation. The use of descriptors and search terms in only English and Portuguese may be another limitation of the study.

### Conclusions

This study protocol presents the main methodological steps that will guide the proposed scoping review and identify and map studies that use spatial analysis as a tool in syphilis-related research in health care. The review may reveal areas with the highest incidence of syphilis, identify which countries most use spatial analysis to study syphilis, and determine the applicability of the technique for syphilis research in each continent.

The results will be published in open access and peer-reviewed journals, favoring the dissemination of knowledge in the scientific community.

## Appendix

Multimedia Appendix 1 Table S1. Complete strategy for searching Medline/Pubmed.

## Abbreviations

BVS: Biblioteca Virtual en Salud
CAPES: Coordenação de Aperfeiçoamento de Pessoal de Nível Superior
CINAHL: Cumulative Index to Nursing and Allied Health Literature
DeCS: Descritores em Ciências da Saúde
JBI: Joanna Briggs Institute
PCC: population, concept, context
PRISMA-ScR: Preferred Reporting Items for Systematic Reviews and Meta-Analyses: Extension for Scoping Reviews
PROSPERO: International Prospective Register of Systematic Reviews
