# Supplementary material for: The Use of Spatial Analysis in Syphilis-Related Research: Protocol for a Scoping Review
Source: JMIR Res Protoc. 2023 Apr 25;12:e43243. doi: 10.2196/43243 (PMC10170366; doi:10.2196/43243)
Supplement: Multimedia Appendix 1 [file resprot_v12i1e43243_app1.docx]

**Multimedia Appendix 1.** Table S1. Complete strategy for searching Medline/Pubmed.

| Strategy | Recovered Studies  (August 30th, 2022 |
| --- | --- |
| (((syphilis[MeSH Terms]) OR ("treponema pallidum"[MeSH Terms])) AND ((((("spatial  analysis"[MeSH Terms]) OR ("geographic information systems"[MeSH Terms])) OR ("disease  hotspot"[MeSH Terms])) OR ("spatio-temporal analysis"[MeSH Terms])) OR ("geographic  mapping"[MeSH Terms]))) OR ((syphilis[Title/Abstract] OR "treponema pallidum"[Title/Abstract]  OR "treponema infections"[Title/Abstract] OR "great pox"[Title/Abstract] OR "early  syphilis"[Title/Abstract] OR lues[Title/Abstract] OR "syphilitic disorder"[Title/Abstract] OR  "venereal syphilis"[Title/Abstract] OR "spirochaeta pallida"[Title/Abstract] OR "treponema  pallida"[Title/Abstract] OR "treponema reiterii"[Title/Abstract]) AND ("spatial  analysis"[Title/Abstract] OR "spatial distribution"[Title/Abstract] OR "spatiotemporal  distribution"[Title/Abstract] OR "spatial analyses"[Title/Abstract] OR "spacial  analysis"[Title/Abstract] OR "spacial analyses"[Title/Abstract] OR kriging[Title/Abstract] OR  krigings[Title/Abstract] OR "spatial interpolation"[Title/Abstract] OR "spatial  interpolations"[Title/Abstract] OR "spatial autocorrelation"[Title/Abstract] OR "spatial  autocorrelations"[Title/Abstract] OR "spatial dependency"[Title/Abstract] OR "spatial  dependencies"[Title/Abstract] OR "kernel density estimation"[Title/Abstract] OR "kernel density  estimations"[Title/Abstract] OR "geographic information system"[Title/Abstract] OR  "geographic information systems"[Title/Abstract] OR "geographic areas"[Title/Abstract] OR  "geographical information system"[Title/Abstract] OR "geographical information  systems"[Title/Abstract] OR "global positioning systems"[Title/Abstract] OR "global positioning  system"[Title/Abstract] OR "GIS platform"[Title/Abstract] OR "geographic  surveillance"[Title/Abstract] OR "disease hotspot"[Title/Abstract] OR "disease  hotspots"[Title/Abstract] OR "burden hotspot"[Title/Abstract] OR "burden  hotspots"[Title/Abstract] OR "disease hot spot"[Title/Abstract] OR "disease hot  spots"[Title/Abstract] OR "disease hotspot burdens"[Title/Abstract] OR "disease spatial  cluster"[Title/Abstract] OR "disease spatial clusters"[Title/Abstract] OR "disease  clusters"[Title/Abstract] OR "disease cluster"[Title/Abstract] OR "disease  clustering"[Title/Abstract] OR "disease clusterings"[Title/Abstract] OR "emergence  hotspot"[Title/Abstract] OR "epidemic hot spot"[Title/Abstract] OR "epidemic  hotspot"[Title/Abstract] OR "epidemiological hot spot"[Title/Abstract] OR "epidemiological  hotspot"[Title/Abstract] OR "transmission hot spot"[Title/Abstract] OR "transmission  hotspot"[Title/Abstract] OR "transmission hotspots"[Title/Abstract] OR "spatiotemporal  analysis"[Title/Abstract] OR "spatio-temporal analysis"[Title/Abstract] OR "space-time  clustering"[Title/Abstract] OR "spatial temporal analysis"[Title/Abstract] OR "spatial  pattern"[Title/Abstract] OR "spatial distribution pattern"[Title/Abstract] OR "spatio temporal  17  analysis"[Title/Abstract] OR "spatio-temporal analyses"[Title/Abstract] OR "spatiotemporal  analyses"[Title/Abstract] OR "spatial temporal analyses"[Title/Abstract] OR "space-time  geography"[Title/Abstract] OR "space time geography"[Title/Abstract] OR "space-time  geographies"[Title/Abstract] OR "geographic mapping"[Title/Abstract] OR "choropleth  mapping"[Title/Abstract] OR "dasymetric mapping"[Title/Abstract] OR "geographical  mapping"[Title/Abstract] OR georeferencing[Title/Abstract] OR "geographic  cartography"[Title/Abstract] OR geocoding[Title/Abstract])) | 112 |
